# Serial Assessment of Hemodynamic and Cerebrovascular Changes After Administration of Mannitol in Postoperative Neurosurgical Patients in the Intensive Care Unit: A Combined Transthoracic and Transcranial Color Doppler Study

**DOI:** 10.7759/cureus.64448

**Published:** 2024-07-13

**Authors:** Soumya Madhusudan, Smita Vimala, Manikandan S, Ayesha Goyal, Yatiraj Singi

**Affiliations:** 1 Anaesthesiology, St. John’s Medical College, Bengaluru, IND; 2 Neuroanaesthesia, Sree Chitra Tirunal Institute for Medical sciences and Technology, Thiruvananthapuram, IND; 3 Anaesthesia, All India Institute of Medical Sciences, Bilaspur, Bilaspur, IND; 4 Forensic Medicine and Toxicology, All India Institute of Medical Sciences, Bilaspur, Bilaspur, IND

**Keywords:** hemodynamics, transcranial doppler, transthoracic echocardiography, cerebral perfusion, intracranial pressure, mannitol

## Abstract

Introduction: Mannitol is widely used in neurosurgical units to mitigate raised intracranial pressure and cerebral edema, crucial in postoperative management. Its hyperosmolar properties reduce brain extracellular fluid, thereby altering cerebral perfusion and cardiac dynamics. However, the temporal and combined effects of mannitol on cardiovascular and cerebrovascular parameters remain inadequately explored in postoperative settings.

Methods: This prospective observational study enrolled 20 adult patients who underwent elective craniotomies for tumor excision. Mannitol was administered to the patients at a dose of 0.5 mg/kg/dose as a bolus dose over 20 to 30 minutes. The time interval was eight hours between the doses (scheduled dosing). Patients received their first dose of mannitol in the ICU after eight hours of intraoperative dose. The patients were given mannitol for two postoperative days and followed up for two days in the postoperative period. Transthoracic echocardiography and transcranial color Doppler were used to assess cardiovascular and cerebrovascular parameters at multiple intervals post-mannitol administration.

Results: Significant increases in mean flow velocities were observed bilaterally immediately post-mannitol administration on the first postoperative day, indicative of improved cerebral blood flow. However, these changes were transient, with no significant variations noted on the second postoperative day. Cerebrovascular resistance, as measured by the pulsatility index, showed non-significant changes bilaterally across both days. Cardiovascular parameters, including stroke volume and cardiac output, remained stable throughout the study period.

Conclusion: Mannitol administration at 0.5 g/kg in postoperative neurosurgical patients transiently improves cerebral perfusion without causing significant hemodynamic instability. This study underscores the importance of monitoring both cerebrovascular and cardiovascular parameters post-mannitol administration to optimize patient management and outcomes.

## Introduction

Mannitol is routinely used in neurosurgical units to reduce intracranial pressure (ICP), a key component of perioperative management. Both experiments and clinical trials have demonstrated that mannitol can attenuate brain edema, decrease ICP, and improve cerebral perfusion. As a hyperosmolar agent, mannitol extracts water from brain extracellular spaces into the intravascular compartment by altering the osmotic pressure gradient between the blood and the brain [1]. This increase in intravascular volume can cause significant changes in cardiac output, stroke volume, and blood pressure and also alter rheology, leading to increased cerebral blood flow (CBF) velocity. Regardless of the underlying pathology causing increased ICP and cerebral edema, mannitol is used to reduce cerebral blood volume.

Major neurosurgical procedures can cause considerable physiological brain insults, resulting in significant morbidity and mortality. Up to 20% of patients who undergo craniotomy develop increased ICP, necessitating the initiation of anti-edema measures in the postoperative period. Owing to its pharmacological properties, mannitol improves cerebral perfusion and oxygenation and reduces edema [2]. Mannitol induces various circulatory effects, such as altering stroke volume and cardiac output. A bolus infusion of mannitol increases cardiac output, arterial pressure, and cerebral perfusion pressure (CPP), with cardiac output rising by up to 30%, thereby increasing CBF [3]. Transient hypotension may occur after bolus administration, while fluid overload and pulmonary edema can manifest as delayed effects. These changes may not significantly impact patients with normal cardiac function but can be detrimental to those with poor cardiac status. Therefore, it is crucial to quantify these changes and take appropriate steps to maintain stable hemodynamics.

Hemodynamic changes induced by mannitol reflect on cerebral hemodynamics. Transient hypotension caused by mannitol can reduce CPP, potentially aggravating secondary brain injuries. Conventional hemodynamic variables, such as blood pressure, heart rate, and central venous pressure, are insensitive and delayed predictors of diminishing circulating blood volume. Studies have reported increases in cardiac output and decreases in total peripheral resistance following mannitol infusion during surgeries [4-5]. However, these are typically single-point measurements with little regard for the time course of these changes.

Sabharwal et al. [6] observed transient increases in stroke volume, cardiac output, and cardiac index after mannitol infusion, and hemodynamic changes correlated with alterations in CBF velocities. Mannitol administration causes cerebral vasoconstriction in distal pial arteries yet improves CBF due to its osmotic properties, which further reduce brain bulk. Wang et al. [7] evaluated the effect of different doses of mannitol on CBF velocities in stroke patients, finding significant improvements in CBF and reductions in ICP in the affected hemisphere. However, this study did not emphasize the cardiovascular effects of mannitol.

While substantial research has evaluated hemodynamic changes after single bolus doses of mannitol in the intraoperative period using thoracic bioimpedance and transesophageal echocardiography, the corresponding changes in cardiac output and stroke volume have not been extensively studied. Similarly, studies employing transcranial color Doppler (TCCD) to assess the effects of mannitol on CBF have not examined the concurrent changes in cardiac parameters. No studies have yet combined these two aspects to determine the temporal effects of mannitol. Thus, there is a lack of comprehensive investigations correlating the cerebral and cardiac effects of mannitol in pathological states, highlighting the need for this study.

This post-doctoral dissertation was uploaded by Sree Chitra Tirunal Institute for Medical Sciences and Technology, Thiruvananthapuram, on the University Repository in 2016-12.

## Materials and methods

This prospective observational study was conducted as a post-doctoral dissertation in the neuro-intensive care unit of the Department of Neuroanesthesia, Sree Chitra Tirunal Institute for Medical Sciences and Technology, Trivandrum, Kerala, India. The study was undertaken with the objective of a serial assessment of the effects of mannitol on cerebral and cardiovascular hemodynamics using TCCD and transthoracic echocardiography (TTE), besides comparing the differences in cardiovascular effects of mannitol on the operated (OP) and non-operated (NOP) sides.

After obtaining approval from the Institutional Ethical Committee (approval letter number: SCT/IEC/843/December-2015 dated March 31, 2016), 25 willing patients posted for elective craniotomy for excision of supratentorial tumors were enrolled in the study after obtaining their informed consent.

Patients aged between 18 and 60 years with American Society of Anesthesiologists (ASA) Grades 1 and 2 who underwent elective craniotomy for the excision of supratentorial tumors and were prescribed mannitol by the operating surgeon were recruited for the study. Unwilling patients, patients posted for emergency surgeries, patients on mechanical ventilation, pregnant and nursing mothers, obese patients with a poor acoustic window, patients with acute neurological deterioration, and those with comorbidities such as anemia, coronary artery disease, renal dysfunction, coagulopathy, infection or sepsis, hemodynamic instability, or requiring either inotrope support or ventriculostomy were excluded from the study.

Patients prescribed mannitol in the postoperative period by the operating neurosurgeon were recruited for the study. All 25 recruited patients received mannitol at a dose of 0.5 mg/kg/dose as a bolus dose over 20 to 30 minutes. The time interval was eight hours between the doses (scheduled dosing). Patients received their first dose of mannitol in the ICU after eight hours of intraoperative dose. The patients were administered mannitol for two postoperative days and were followed up for two days in the postoperative period.

TCCD and TTE variables were recorded at the time intervals of 5, 15, 30, 45, and 60 minutes after the first dose of mannitol administration on postoperative day 1 (D1) and postoperative day 2 (D2). Recordings were obtained from both the OP and the NOP.

TCCD measurements

A 2 Mhz transducer probe was used to insonate the middle cerebral artery (MCA) through the transtemporal window. MCA was identified at a depth of 55 to 65 mm with a flow toward the probe. MCA was insonated on both the OP and NOP sides.

The normal spectral waveform shows peak systolic velocity (PSV) and end-diastolic volume (EDV). On this spectral waveform, the mean expiratory flow (mFV) is derived by applying the formula mFV = EDV+1/3(PSV-EDV).

The pulsatility index (PI) of Gosling determines cerebrovascular flow resistance. It is derived from the difference between PSV and EDV divided by mFV. PI is independent of the angle of insonation, and a value greater than 1.2 represents increased CBF. A significant correlation exists between PI and ICP. An increase in ICP will reduce the compliance of the cerebrovascular system and augment velocity variation, increasing the denominator mFV. Hence, PI is extremely sensitive to ICP changes and can be used as a surrogate marker of ICP. ICP is derived from PI using the formula ICP=11.1xPI-1.43.

TTE measurements

TTE was performed using a 3-5 Mhz probe on patients in the supine position. For the parasternal long-axis view, the probe is placed 2 to 3 inches left of the sternum at the fourth or fifth intercostal space. Thereafter, an apical four-chamber view is acquired to trace the left ventricle endocardial border at the end-systolic and end-diastolic phases of the same cardiac cycle. Stroke volume is measured from the left ventricular outflow tract (LVOT) X velocity time integral (VTI). Cardiac output is derived from stroke volume multiplied by heart rate.

Statistical analysis

The collected data was tabulated into the data processing software (Microsoft Excel 2019, Microsoft Corporation, Washington, USA) and analyzed using SPSS Statistics version 17 (SPSS Inc. Released 2008. SPSS Statistics for Windows, Version 17.0. Chicago: SPSS Inc.). Results obtained are expressed as mean + SD. A paired t-test was applied to compare the values of the OP and NOP sides. A repeated measures ANOVA was used to compare the data of the OP and NOP sides on D1 and D2. A p-value less than 0.05 was considered statistically significant.

## Results

A total of 25 patients were enrolled in the study, of whom three had poor acoustic windows and two underwent re-exploration. The study cohort finally consisted of 20 patients in a ratio of 2:3 male-female. The patient characteristics for the 20 patients are presented in Table 1.

**Table 1:** Characteristics of the patients COPD: chronic obstructive pulmonary disease, CKD: chronic kidney disease, ITP: idiopathic thrombocytopenic purpura

Patient characteristics	N	%
Age		
18-30 yrs	7	35
31-40 yrs	2	10
41-50 yrs	6	30
51-60 yrs	5	25
Gender		
Male	8	40
Female	12	60
Comorbidities		
Hypertension	5	25
Diabetes mellitus	4	20
Bronchial asthma	2	10
COPD	1	5
CKD	1	5
ITP	1	5
Seizure disorders	1	5
Nil	7	35
Tumor decompression surgical approach		
Pterional craniotomy	10	50
Bifrontal craniotomy	5	25
Parietooccipetal craniotomy	2	10
Unilateral frontal craniotomy	2	10
Basifrontal approach (Poppins method)	1	5

The comparison of mFV through MCA on the OP and NOP sides of D1 was statistically significant. mFv starts increasing at 5 minutes on the OP side (p<0.001), which is a statistically significant increase compared to the NOP side (Table 2). A repeated measure ANOVA shows F=175 and p≤0.001 on the OP side, whereas it was F=76.829 and p<0.00132 on the NOP side.

**Table 2:** Comparing mFV between the OP and NOP sides on D1 mFV: mean expiratory flow (cm/sec), OP: operated, NOP: non-operated, D1: postoperative day 1

	OP side		NOP side		Paired test	
	Mean	SD	Mean	SD	t	p
Baseline	49.2	9.2	50.8	9.3	-0.944	0.357
After 5 mins	86.1	14.8	53.3	13.8	17.139	<0.001
After 15mins	135.4	24.4	50.9	13.8	17.399	<0.001
After 30mins	80.6	11.0	107.9	22.7	-21.176	<0.001
After 45 mins	83.3	14.0	108.3	23.9	-7.536	<0.001
After 60 mins	139.7	24.8	107.7	25.0	5817	<0.001

The comparison of mFV through MCA on D2 after mannitol administration between OP and NOP was statistically insignificant, and similarly, there was no statistically significant increase observed after mannitol administration on D2 (Table 3).

**Table 3:** Comparing mFV between the OP and NOP sides on D2 mFV: mean expiratory flow (cm/sec), OP: operated, NOP: non-operated, D2: postoperative day 2

	OP side		NOP side		Paired test	
	Mean	SD	Mean	SD	t	p
Baseline	51.6	10.3	57.4	16.6	-1.707	0.104
After 5 mins	58.2	16.9	55.7	12.3	-0.550	0.589
After 15 mins	52.0	11.0	52.9	7.9	-0.480	0.637
After 30 mins	50.8	10.0	53.9	14.2	-0.902	0.379
After 45 mins	50.8	10.0	55.6	9.2	-1.793	0.089
After 60 mins	52.9	13.0	52.8	11.7	0.038	0.970

**Figure 1:**
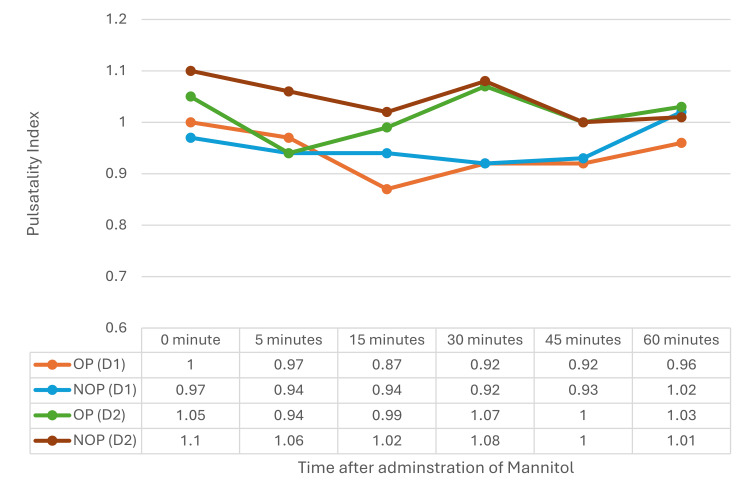
PI on both OP and NOP sides on D1 and D2 PI: pulsatility index, OP: operated, NOP: non-operated, D1: postoperative day 1, D2: postoperative day 2

PI on D1 showed an insignificant decrease from baseline, whereas no such change was seen on D2. The comparison of PI on the OP and NOP sides on D1 and D2 was not statistically significant (Figure 1).

**Figure 2:**
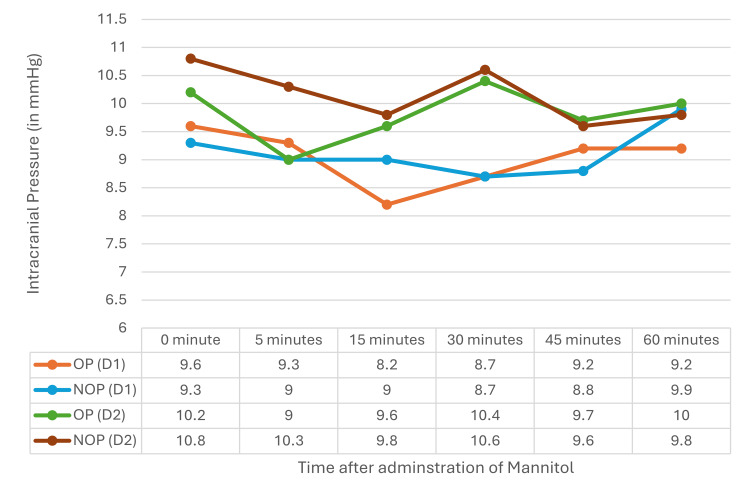
ICP on both OP and NOP sides on D1 and D2 ICP: intracranial pressure, PI: pulsatility index, OP: operated, NOP: non-operated, D1: postoperative day 1, D2: postoperative day 2

The estimated ICP shows variations on D1, whereas the ICP was stable on D2. The variation in ICP was statistically insignificant. The comparison of ICP on the OP and NOP sides on D1 and D2 was not statistically significant (Figure 2).

**Figure 3:**
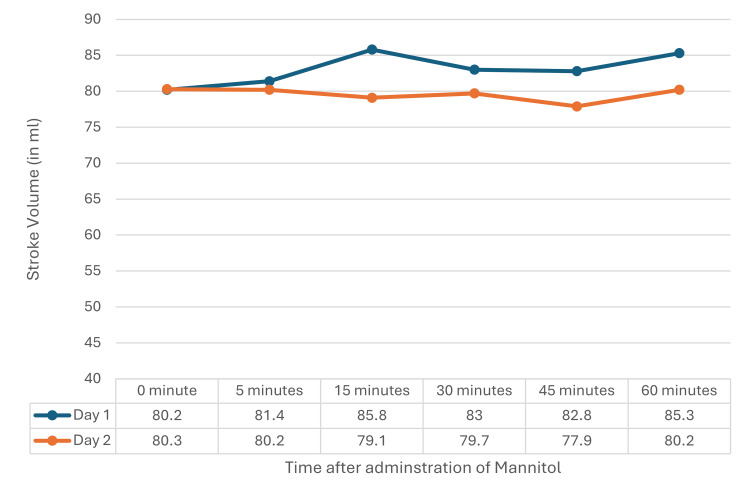
Stroke volume on D1 and D2 A repeated measure ANOVA did not show a statistically significant difference between D1 (F=0.856, p=0.514) and D2 (F=0.150, p=0.980). ANOVA: analysis of variance, D1: postoperative day 1, D2: postoperative day 2

**Figure 4:**
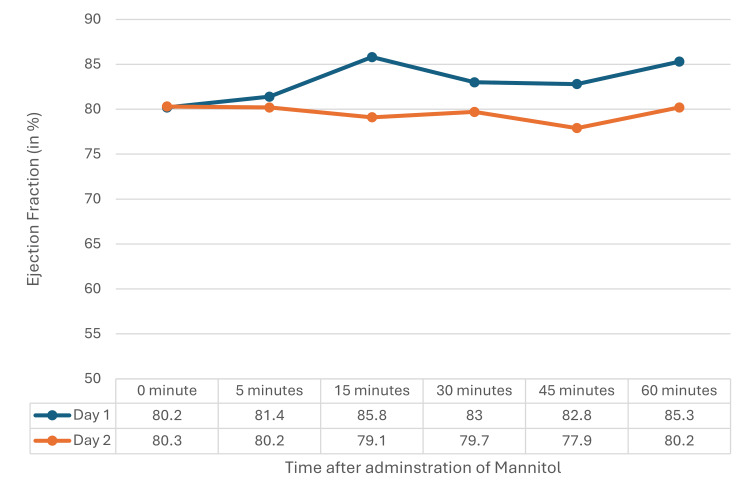
Ejection fraction on D1 and D2 A repeated measure ANOVA did not show a statistically significant difference between D1 (F=1.608, p=0.165) and D2 (F=0.114, p=0.989). ANOVA: analysis of variance, D1: postoperative day 1, D2: postoperative day 2

**Figure 5:**
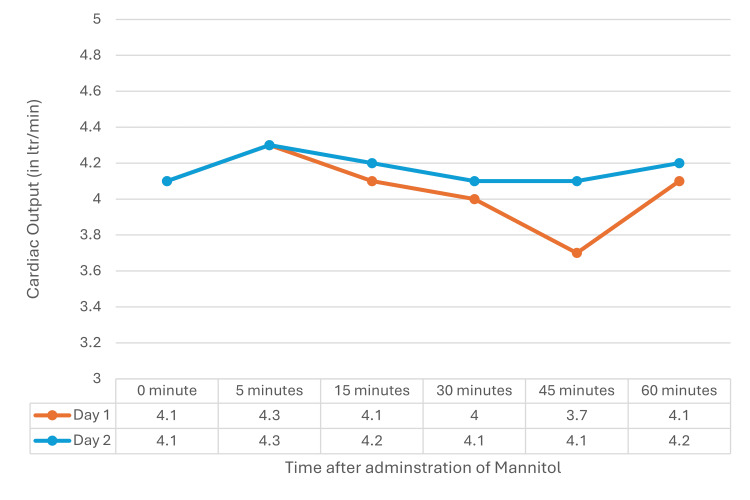
Cardiac output on D1 and D2 Repeated measure ANOVA did not show a statistically significant difference between D1 (F=1.253, p=0.291) and D2 (F=1.143, p=0.982). ANOVA: analysis of variance, D1: postoperative day 1, D2: postoperative day 2

The cardiovascular parameters obtained on TTE, i.e., stroke volume, ejection fraction, and cardiac output, were stable without any major changes on D1 and D2 after mannitol administration. The variation in readings at any point in time on D1 and D2 did not have any statistical significance (Figures 3-5).

**Figure 6:**
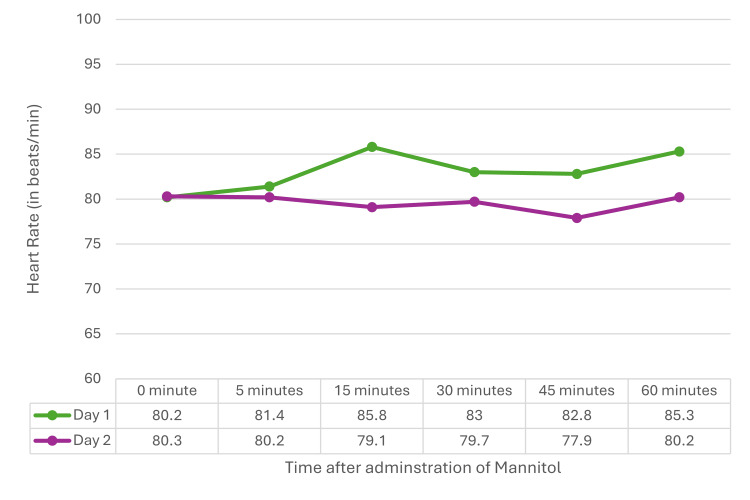
Heart rate on D1 and D2 Repeated measure ANOVA did not show a statistically significant difference between D1 (F=1.044, p=0.396) and D2 (F=0.566, p=0.726). ANOVA: analysis of variance, D1: postoperative day 1, D2: postoperative day 2

**Figure 7:**
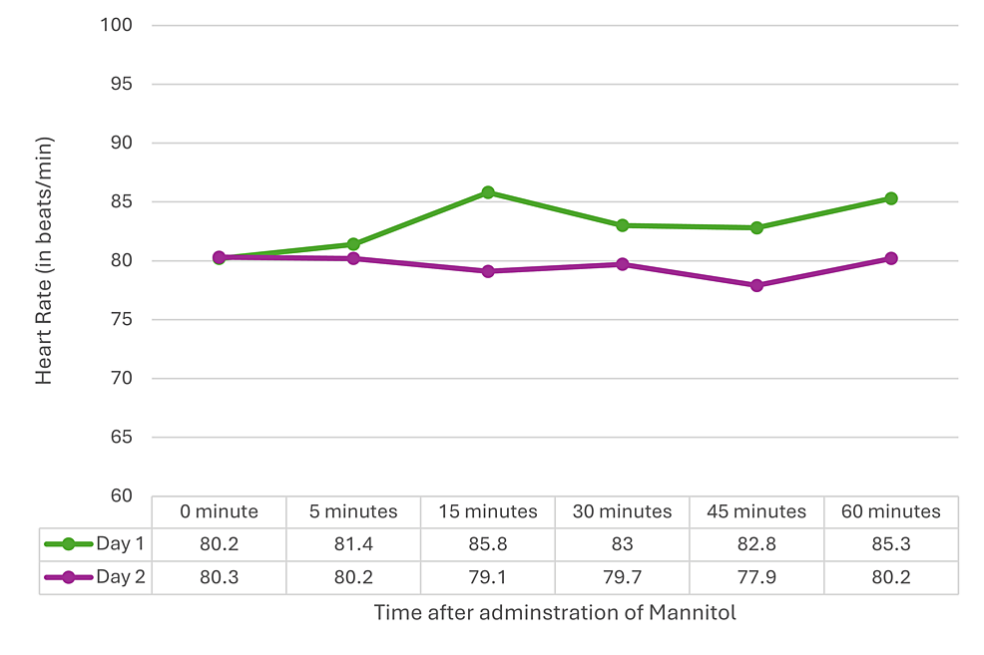
Mean arterial pressure on D1 and D2 Repeated measure ANOVA noticed no significant difference between D1 (F=2.26, p=0.508) and D2 (F=0.583, p=0.713). ANOVA: analysis of variance, D1: postoperative day 1, D2: postoperative day 2

The vital hemodynamic parameters such as heart rate and mean arterial pressure were measured at baseline, 5, 15, 30, 45, and 60 minutes after mannitol administration on D1 and D2. The heart rate and mean arterial pressure did not show any statistically significant changes at all the time intervals on both D1 and D2 (Figures 6-7).

## Discussion

The current study observed significant changes in mFV on both the OP and NOP sides following mannitol administration. On the D1, mFV increased within five minutes on both sides, with a more significant change on the OP side within the first 15 minutes, followed by a brief decrease at 30 and 45 minutes. By 60 minutes, a second peak in mFV was observed on the OP side, while the NOP side showed a gradual increase from 30 to 60 minutes. Bolus doses of mannitol increased serum osmolarity, transiently increased blood volume, decreased blood viscosity, enhanced red cell deformability, and increased CBF. This increase in cerebral perfusion leads to an increase in mFV and reduces further secondary damage to the brain [8-10]. The initial rise in mFV on the OP side can be attributed to defective autoregulation, leading to increased CBF immediately after mannitol administration, which was not observed on the NOP side. The second peak in mFV aligns with the osmotic action of mannitol and improved rheology.

Wang et al. [7] found similar increases in mFV in both hemispheres from 30 to 120 minutes with different doses of mannitol, while Vicenzini et al. [11] noted higher MCA flow velocity on the affected side during and after mannitol infusion, attributed to reduced perilesional edema. However, the biphasic response noted in the current study was absent in theirs. On D2, no significant changes in mFV were observed, suggesting a return to cerebral homeostasis. Mannitol reduces ICP, and the PI measured by TCCD can reflect this reduction. The current study showed a gradual decrease in PI on both sides, indicating reduced cerebrovascular resistance and improved perfusion. Similar PI reductions were observed by Wang et al. [7], while Vicenzini et al. [11] reported no PI changes due to preserved pulsatility. ICP derived from PI showed a gradual but non-significant decrease. Similarly, Kirkpatrick et al. [12] reported a reduction in diastolic flow following mannitol administration, but no statistically significant decrease in PI was noted.

Repeated mannitol administration did not alter mean arterial pressure on either day. Previous studies by Diringer et al. [13] and Chatterjee et al. [14] also observed no consistent changes in blood pressure or heart rate after mannitol administration. However, Gilsanz et al. [15] found significant postoperative hypotension in patients due to hypovolemia, which can be exacerbated by mannitol, leading to adverse outcomes such as renal failure, pulmonary edema, and electrolyte imbalances. Despite its benefits, mannitol does not necessarily lead to favorable clinical outcomes, as highlighted by Cochrane Reviews on mannitol for head injury and stroke. Earlier studies, such as Mendelow et al. [16], showed increased CBF after 2 g/kg mannitol administration in head injury patients, with greater increases in less damaged hemispheres. Muizelaar et al. [17] noted that mannitol increased CBF in patients with defective autoregulation and low basal CBF, whereas Sharma et al. [18] observed impaired autoregulation post-supratentorial craniotomies, likely influencing our findings.

In the current study, no significant changes in left ventricular systolic function, stroke volume, cardiac output, or ejection fraction were observed post-mannitol administration. Chatterjee et al. [14] reported increases in end-diastolic area, cardiac output, and stroke volume with higher mannitol doses, which were not seen in the current study. Sabharwal et al. [6] observed hemodynamic changes during craniotomy with larger mannitol doses, differences likely due to larger doses and intraoperative factors absent in the current postoperative study.

A large dose of mannitol reduces intravascular volume, leading to hypotension, whereas in some cases, it can increase intravascular volume and lead to congestive cardiac failure. Animal studies have shown that mannitol can leak into brain tissue across the disrupted blood-brain barrier, leading to a rebound increase in ICP [19]. For patients with consistently prolonged high ICP, careful weaning of hyperosmolar agents is needed to prevent this rebound phenomenon [20]. The smallest dose of mannitol should be chosen, guided by ICP monitoring and the clinical assessment of the patient [21].

In the present study, it was observed that mannitol administration impacts both cerebral and cardiovascular dynamics. Careful consideration of dose and timing is crucial, especially in patients with cardiac conditions, to prevent adverse outcomes and ensure effective treatment. According to the Brain Trauma Foundation Guidelines Task Force, mannitol should control ICP at doses between 0.25 and 1 g/kg body weight in a single dose, administered over 20-30 minutes [22]. No evidence exists for repeated administration or continuous infusion, and limited data exists for administration over several days with no clear benefit [23].

Limitations

A significant limitation of this study is the lack of direct comparison between invasive and non-invasive techniques for measuring CPP, CBF, and CO. Invasive monitoring methods like ICP and pulmonary artery catheterization are not warranted in low-risk groups (ASA 1 and 2). While TCCD can only be performed intermittently, ICP catheters provide real-time monitoring. TCCD, being operator-dependent, is prone to technical errors and inter-observer variability. However, extensive case series support TCCD-derived indices as valid surrogate markers of cerebrovascular homeostasis compared to gold standard ICP measurements. Additionally, the number of participants was limited due to the nature of the surgeries and the surgeon's discretion in using mannitol. Thus, it is difficult to generalize the results of the study to other pathologies, like trauma or stroke. Additionally, the absence of cerebral autoregulation assessment and the lack of outcome analysis regarding mannitol's effect on clinical improvement further limit the study's robustness.

Thus, future studies with larger sample sizes and addressing the limitations of this study need to be undertaken to better analyze the effects of mannitol on central nervous and cardiovascular hemodynamics.

## Conclusions

Mannitol, introduced in 1960, remains a crucial osmotherapy agent for traumatic brain injury patients, particularly in pre-hospital care, herniation treatment, and intraoperative brain relaxation. The current study found that administering 0.5 g/kg of mannitol three times daily significantly improved cerebral perfusion without causing ICP rebound or significant cardiovascular changes in D1; however, these desired effects were not evident in D2. Therefore, it is safe to suggest that mannitol can be safely used to control ICP and reduce secondary brain injury, but at the same time, it's not prudent to administer mannitol without clinical signs of raised ICP. Moreover, instead of invasive methods of ICP and hemodynamic monitoring like intraventricular catheter placement and intraparenchymal probes, which are not feasible for routine clinical practice, the use of non-invasive techniques like TCCD and TTE can be explored.
